# Uptake of health insurance by the rural poor in Ghana: determinants and implications for policy

**DOI:** 10.11604/pamj.2018.31.124.16265

**Published:** 2018-10-19

**Authors:** Aaron Alesane, Benjamin Tetteh Anang

**Affiliations:** 1 School of Agriculture, Policy and Development, University of Reading, Reading, Berkshire, England; 2 Department of Agricultural Economics and Extension, University for Development Studies, Tamale, Ghana

**Keywords:** Health insurance, logit model, rural poor, Ghana

## Abstract

**Introduction:**

Financing access to healthcare services in developing countries remains a major challenge despite recent advances towards implementation of various health insurance policies in many low and middle-income countries. The use of health insurance is considered an important means to achieve universal health coverage. However, uptake of health insurance in most developing countries remains low as a result of several challenges. Empirical evidence of factors restraining enrolment is rare in many developing countries including Ghana. This paper therefore sought to investigate the factors associated with the uptake of health insurance products and the implications thereof for policy, using Awutu Senya West District of Ghana as case study.

**Methods:**

A logit model was used to analyze data from 178 respondents randomly selected from two microfinance groups operating in the study area.

**Results:**

The results indicate that insurance uptake is higher among younger people, but lower among women. Older women are however more likely to take up health insurance compared to older men. In addition, the study reveals that insurance uptake increases with level of education but decreases with household size.

**Conclusion:**

The study concludes that even though the premium on health insurance coverage in Ghana is arguably low, socio-demographic characteristics such as age, sex, literacy level and household size affect the decision to enroll. Adequate public sensitization on the benefits of the scheme and decreasing the statutory age for exemption from premium payment, especially in rural localities, are some of the measures suggested to enhance health insurance uptake in Ghana and other developing countries.

## Introduction

The cost of providing healthcare services is one of the growing concerns and major budgetary challenges facing most countries [1, 2]. Expenditure on healthcare services remains a large proportion of household incomes in many developing countries with many poor households unable to pay for sudden unexpected healthcare bills due to high incidence of poverty and low incomes. Many people in rural communities rely on traditional sources in addressing their healthcare needs due to inaccessibility to and high cost of orthodox healthcare services. Avoidance of catastrophic health expenditures and the need to strive towards universal health coverage have called for the need to develop models of health insurance to tackle these challenges [3-5]. Consequently, governments in many developing countries have resorted to health insurance schemes that seek to pool resources in order to offer subsidized healthcare services for their populations. Health insurance typically refers to insurance cover against the risk of personally incurring medical expenses. The provision of subsidized and affordable health insurance coverage is essential for the attainment of universal health coverage, which is an important goal set forth under the Sustainable Development Goals (SDGs). Recognizing the importance of a healthy population in national development and the impelling social obligation to provide universal health coverage for its citizens, the government of Ghana in 2003 put in place a system of health insurance scheme that subsidizes the cost of basic healthcare services for its citizenry. This made Ghana the first country in sub-Saharan Africa to institute a National Health Insurance Scheme (NHIS) to enroll its citizens onto health insurance [1]. The institution of the NHIS was an effort by the government of Ghana to accelerate the attainment of universal health coverage. The National Health Insurance Scheme seeks to avert the burden of out-of-pocket payment for healthcare services through a system of pooling resources to finance health expenditures of the insured. The NHIS is a form of national health insurance and seeks to provide equitable access and financial coverage for basic healthcare services. The scheme is operational across the country and participation is voluntary as with any insurance policy. To improve efficiency of operation and accessibility to healthcare services, District-level Health Insurance Schemes have been established. Insurance cover under the National Health Insurance Scheme (NHIS), which runs concurrently with a cash-and-carry system that requires out-of-pocket payments for the uninsured, has been recognized as a successful social intervention mechanism despite some challenges confronting the scheme. A one-time premium payment was proposed by the erstwhile National Democratic Congress (NDC) government in 2009, but the proposal did not go beyond rhetoric. Currently, the scheme operates through the payment of a yearly premium, with the elderly and indigent populations receiving free enrolment onto the scheme.

Despite the general acceptability of health insurance as a means to attaining universal health coverage, uptake of health insurance has not reached desired expectations in most countries, including Ghana [2]. A number of studies have been carried out in many countries in an attempt to gain a better understanding of the factors limiting uptake of health insurance, especially in developing countries [6-11]. Understanding the factors inhibiting health insurance uptake will help policy makers to design more effective health insurance schemes to ensure attainment of universal health coverage. Empirical evidence of the factors influencing health insurance uptake indicate that age plays an important role in enrolment. Studies by [6] indicate that health insurance cover increases with age. This was attributed to increment in additional healthcare needs and increased financial security of older people. This finding is supported by other studies [8, 12]. Other empirical studies show a significant difference in health insurance coverage based on gender. Other studies indicate lower ownership of health insurance cover by men because they are perceived to be risk-takers while women have higher health insurance coverage due to their greater need for more health services [8, 12]. However, [6] found no significant difference in health insurance coverage based on gender differences. A number of empirical studies suggest a positive association between health insurance coverage and the level of education, attributable to higher purchasing power and access to information on health insurance [8, 13, 14]. A study by [7] found that possessing tertiary education is positively related to health insurance uptake in rural Kenya. Education enhances the health seeking behavior of individuals, thus influencing insurance uptake. Household size also influences health insurance uptake according to the extant literature. A positive association between household size and uptake of health insurance was observed by [13]. On the other hand, [8] and [15] observed an inverse relationship between household size and health insurance coverage in Kenya and Nigeria respectively. Additional household members exert financial strain on the household which may lead to lower insurance coverage. While [7] did not find any relationship between household size and health insurance coverage in Kenya, [6] observed that averaged sized households in Kenya were more likely to enroll in health insurance compared to smaller and larger households. Furthermore, according to [7], having knowledge of the benefits of health insurance is positively related to health insurance uptake. Knowledge of the benefits of health insurance is influenced by educational level, accessibility to health extension services, proximity to health facilities and the availability of qualified health personnel. Majority of the population in developing countries are rural hence the likelihood of many people being unaware of the benefits of certain government programmes and policies cannot be ruled out.

Additionally, [7, 9, 16] respectively identified higher uptake of health insurance among married women in Kenya, Ghana and South Africa. These studies associated higher insurance uptake of married couples to pooling of financial resources leading to increased income and hence, the ability to afford health insurance cover. In another study, [6] observed that the likelihood for married patients in Kenya to take up health insurance cover was 10 times higher than unmarried patients. Reasons given by the authors included the avoidance of catastrophic health expenditure, the increased collective pooled income of the spouse and the desire to insure children. In another study, [8] argued that some employers provide insurance cover for spouses and children accounting for the increased likelihood of enrolment through the spouse's insurance cover. A positive association between being married and possessing health insurance was also reported by [15] and [17]. Furthermore, [8] observed that more educated women have a higher likelihood to take up health insurance. This indicates that provision of education to women is likely to spur health insurance uptake in developing countries. Other factors affecting insurance uptake include employment status [18-20] and level of income. A positive relationship between household income and health insurance uptake was observed by [10, 11] in their studies on health insurance uptake in Ghana. Despite the numerous benefits associated with enrolment in the national health insurance scheme (NHIS) and efforts by the scheme providers to ensure high enrolment rates, studies indicate that many Ghanaians are not enrolled. For instance, although indigents are entitled to free enrolment in the national health insurance scheme, [2] observed that procedures for the identification of the poor indigents remain ineffective thereby excluding many from receiving free health care. Consequently, the number of indigents enrolled in the NHIS continues to decline over the years. Also, economic and financial barriers have led to NHIS membership that is skewed against the poor and marginalized. It is in the light of the foregoing that this study was carried out to investigate the factors affecting enrolment onto the health insurance schemes, especially by the rural poor in Awutu Senya West District of Ghana, and the implications of the research findings to health policy in Ghana. The study solicited responses from participants in two microfinance groups operating in the study area. The data was subjected to econometric analysis in order to draw conclusions.

## Methods

**Figure 1:**
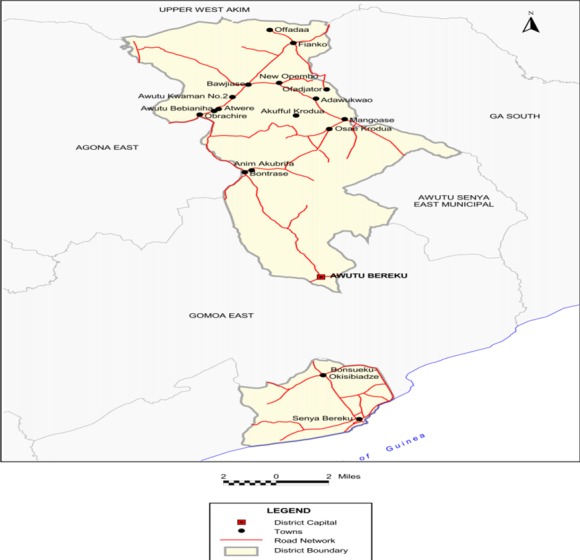
Study area showing district map of Awutu Senya West

**Study area:** The study was carried out in Awutu Senya West District in the Central Region of Ghana. The District is bordered to the West by Effutu Municipal, to the East and North by Awutu-Senya East District, and to the South by the Gulf of Guinea. The 2010 population census gave an estimated population of 86,884 with 45,981 female and 40,903 male. Fishing, food and cash crop production, trading, arts and craft, are important economic activities in the District. A map of the study area is shown in Figure 1.

**Sampling and data:** Participants for the study were selected from two microfinance groups operating in the District. These microfinance groups represented two distinct microfinance models: Credit with Education (CwE) and Village Savings and Loans (VSL). The Credit with Education (CwE) microfinance model is based on members making a financial commitment to a formal financial institution (i.e. a Rural Bank) and to a group. Members save with the rural bank via the groups and then obtain loanable funds from it for on-lending to members. The second model, the Village Savings and Loans (VSL) is based on resource pooling through share acquisition by members which forms loanable funds for members. Dividends are paid to members in proportion to shares bought from the agreed-upon interest payments on loans taken. At the time of the study, the VSL comprised 115 groups while the CwE had 250 groups. Membership in each group comprised approximately 30 members. A total of 202 respondents were randomly selected for the study, made up of 101 VSL members and 101 CwE members. A semi-structured questionnaire was used as the data collection tool. However, due to incomplete data, 178 respondents were included in the final analysis. Data collected included socio-economic and demographic characteristics of the respondents such as age, sex, educational level, marital status, household size and amount of savings.

**Empirical logit model:** Uptake of health insurance policy is a binary decision involving the choice to either enroll or abstain, hence the application of a binary choice model to analyze the decision. Probit and logit models are two of the appropriate and commonly used frameworks for analyzing such decisions because of their suitability in dealing with decisions involving a binary choice. Probit and logit models provide similar estimates and the choice one often depend on the preference of the author. This study used the logit model because of the easy interpretation of the estimates. Furthermore, several authors have used the logit model to investigate binary decisions in other studies. In the case of binary choice model, it is assumed that the response variable (y_i_= insurance uptake) takes the value y=1 for individuals with insurance and y = 0 for those without insurance. There is however an underlying unobserved latent variable y_i_* which defines the probability of participation in health insurance. The latent variable is defined as

$$y_{i}^{*}=\beta x_{i}+\varepsilon_{i}$$

where x_i_ is a vector of explanatory variables determining y_i_*, β is a vector of unknown parameters and ε_i_ is the random error term. The unobserved latent variable is related to the observed response variable as follows:

$$y_{i}=\left\{ \begin{array}{l} 1\;\;if\;y_{i}^{*}>0\\ 0\;\;if\;y_{i}^{*}\geq 0 \end{array}\right.$$

The empirical logit model for the study was specified as follows:

$$y_{i}^{*}=\beta_{0}+\beta_{1}SEX_{\text{i}}+\beta_{2}EDUC_{\text{i}}+\beta_{3}AGE_{\text{i}}+\beta_{4}AGESQD_{\text{i}}+\beta_{5}MARRIED_{\text{i}}+\beta_{6}SAVINGS_{\text{i}}+\beta_{7}HHSIZE_{\text{i}}\beta_{8}AGE*SEX_{\text{i}}+\varepsilon_{\text{i}}$$

where y_i_* represents a latent continuous variable for uptake of health insurance, such that y=1 if y_i_*>0, and y=0 if y_i_*≤0. β is a vector of unknown parameters to be estimated and ε_i_ is the random error term. The explanatory variables in the logit model include sex of the respondent (SEX), educational level (EDUC), respondent's age (AGE), the squared valued of respondent's age (AGESQD), marital status (MARRIED), amount of current savings (SAVINGS), household size (HHSIZE) and the interaction term for age and sex (AGE*SEX).

## Results

**Characteristics of the respondents:** The descriptive characteristics of the respondents in the study are provided in Table 1. The average age of the respondents is 42 years. On average, respondents had 2 years of formal education, 5 household members, and GH¢163 total savings (approximately US$37). Majority of the respondents were female (77%), while 62% were married. The characteristics of the respondents according to their participation in health insurance are presented in Table 2. Out of the 178 respondents, 34% had health insurance cover. The insured were statistically younger and had more education and smaller household size. The level of education of the respondents was very low, at less than two years of formal education. Majority of the respondents are uneducated people, which is typical of rural communities. In terms of gender, close to 38% of the insured were female compared to 15% for the uninsured. The insured had higher personal savings than the uninsured but there was no significant difference between the amounts saved.

**Table 1:** Data description and summary statistics of the respondents

Variable	Definition	Mean	SD	Min	Max
Age	Age of household head (years)	41.84	9.417	19	65
Sex	Sex of household head (1 = male)	0.230	0.422	0	1
Married	Marital status (1 = married)	0.618	0.487	0	1
Education	Education of household head (years)	1.680	0.525	1	3
Household size	Household size (number)	5.124	2.296	1	15
Savings	Amount of savings (GH¢)	163.3	111.5	10	700

**Table 2:** Summary statistics of the insured and uninsured

Variable	Insured (*n* = 61)		Uninsured (*n* = 117)		*t*-test^Ϯ^
	Mean	SD	Mean	SD	
Age	40.08	10.17	42.75	8.908	1.807*;
Sex	0.377	0.489	0.154	0.362	-3.449***
Marital status	0.607	0.493	0.624	0.486	0.2252
Education	1.852	0.511	1.590	0.511	-3.255***
Household size	4.443	2.377	5.479	2.180	2.917***
Savings	176.2	108.2	156.5	113.0	-1.116

Ϯ signifies the test of difference in means between insured and uninsured. ***, ** and * stand for statistical significance at 1, 5 and 10 percent level, respectively

**Determinants of health insurance uptake:** The main objective of the study was to estimate the predictors of health insurance uptake among the sampled respondents. Maximum likelihood estimates of the logit model of insurance uptake among respondents in the sample are presented in Table 3. The predictors of health insurance uptake included age, sex, education, household size, and the interaction term for age and sex. From Table 2, insurance uptake is lower among older people as indicated by the negative and significant coefficient of the age variable. A unit increase in the age of the respondent decreases the probability of taking up insurance by 0.08. However, with an increase in age above a certain threshold, insurance uptake begins to increase. In other words, insurance uptake initially decreases with age but increases with advancement in age. The study finds lower participation of women in health insurance in Ghana. The coefficient of the gender variable is positive and statistically significant at 1% level indicating higher insurance uptake by male respondents. Being male increases the probability of taking up insurance by 0.93. The interaction of age and gender showed that older women were more likely to take up insurance compared to older men. This is reflected in the positive coefficient which is significant at 5% level. The probability of older women taking up health insurance is 0.04 higher than older men. The level of education is an important variable in most studies on uptake of health insurance. The result of this study indicates that health insurance uptake positively correlates with level of education. The coefficient of the education variable is significant at 1% level. Hence, an increase in the level of education is associated with an increase in health insurance uptake. An additional year of education increases the probability of taking up insurance by 0.26. Furthermore, the results of the study reveal that insurance uptake decreased with household size. The coefficient of the household variable is significant at 10% level. The marginal effect estimate indicates that addition of one member to the family decreases the probability of taking up insurance by 0.04.

**Table 3:** Maximum likelihood estimates of the logit model of insurance uptake

Variable	Coefficient	Std. Error	*P* – value	Marg. Eff.
Age	-0.366**	0.157	0.019	-0.079
Age squared	0.005**	0.002	0.013	0.001
Sex	8.060***	2.734	0.003	0.928
Marital status	-0.229	0.446	0.608	-0.050
Education	1.189***	0.417	0.004	0.258
Household size	-0.180*	0.092	0.052	-0.039
Age*Sex	-0.164**	0.066	0.013	-0.036
Savings	0.116	0.284	0.681	0.025
Constant	4.479	3.063	0.144	-
Log-likelihood	-94.22			
LR chi2 (8)	40.41***			
Percentage correctly classified	76.97			
Pseudo R2	0.177			

***, ** and * stand for statistical significance at 1, 5 and 10 percent level, respectively

## Discussion

**Characteristics of the respondents:** Most rural communities in Ghana are characterized by low level of education as reflected in this study. This result is consistent with the findings of [21]. Education plays an important role in economic growth because of its positive effect on human capital development. The low level of education among the respondents is therefore likely to have adverse effects on several economic outcomes and the quality of individual and intra-household decisions. Several research studies have shown that poor people save less as a result of low incomes and participation in low-income jobs due to low capabilities [22, 23]. This is reflected in the small absolute amounts of savings by the respondents in this study. The amount of saving was used as a proxy for respondents' level of income which impacts levels of health insurance uptake.

**Determinants of health insurance uptake:** Many studies have reported a positive association between age and insurance uptake [6, 8, 12] without consideration of the life cycle effect. Utility maximization and choices of individuals change over their life cycle. Hence, by including the quadratic term of the age variable in the analysis, the life cycle effect of increment in age is captured and provides a better picture of how individuals behave in their decisions regarding uptake of health insurance. Thus as shown in this study, insurance uptake initially decreases with age but beyond a certain threshold, insurance uptake then increases with age. A likely reason for this phenomenon is that younger folks may not have additional family responsibilities broadly and healthcare needs of children particularly, whereas at advanced ages, there is usually depreciation of inherited health stock resulting in additional costs from healthcare needs (hence the greater demand for insurance). The direction of influence of gender on health insurance uptake is varied in the literature. A study by [6] in Kenya did not find any significant difference in health insurance uptake on the basis of respondent's gender. However, some studies have indicated lower insurance uptake by men because they are perceived to be risk-takers while other studies have indicated higher health insurance coverage by women due to their greater need for more healthcare services [8, 12]. The lower participation of women in health insurance in Ghana may be attributed to the low economic and social position of women relative to men. Women are more economically-disadvantaged and usually have lower access to interventions and programs due to the highly patriarchal nature of most rural communities. The level of education of women is also usually low which results in lower female participation in social interventions such as health insurance. It is also possible that women seek more alternative healthcare services away from the orthodox health system.

The interaction of age and gender indicates that policy recommendations based on the effect of a single factor variable on an outcome variable of interest may yield results that are not exhaustive. Several factors interact to influence individual choices and preferences, and the inclusion of interaction terms in econometric analysis is likely to provide more detailed and reliable results. As shown by the findings of this study, whereas insurance uptake is lower for female respondents, uptake is higher for older women. The higher participation of older women in health insurance compared to older men does not render an easy interpretation. However, the result suggests that as women grow older they place higher premium on their healthcare needs more than older men do. The result is consistent with a priori expectation because of the role education plays in enhancing quality decision-making. Education improves the health seeking behavior of individuals. In other words, educated people are more able to acquire and process information leading to informed decisions that enhance their well-being. Education potentially also increases purchasing power and access to health insurance information resulting in higher insurance uptake by educated people. Similar results have been obtained by [8, 13, 24]. The relationship between household size and insurance uptake implies that larger households are less likely to have insurance cover. As the household increases in size, the available resources are strained so that there is little money laid aside to cater for health insurance. The result agrees with [8, 15] in their study of factors influencing health insurance uptake in Nigeria and Kenya respectively. Also, [25] found household size to be negatively related with health insurance uptake in Kenya. In other studies, [8] observed higher health insurance coverage among average sized households in Kenya while [7] found no relationship between household size and health insurance uptake in rural Kenya.

## Conclusion

The study sought to examine the predictors of health insurance uptake by rural dwellers using the Awutu Senya West District of Ghana as case study. Using a multivariate logit model, the study identified the following as determinants of health insurance uptake: respondent's age, sex, educational level as well as household size. The following conclusions emerge from the findings of the study: 1) Insurance uptake is lower among older people but increases at a certain threshold. 2) Insurance uptake is lower for female respondents as compared to males. 3) The interaction of age and gender however shows that older women are more likely to take up insurance compared to older men. 4) Education enhances uptake of insurance. 5) Household size is negatively associated with insurance uptake. The findings of the study have the following implications for policy: Health insurance uptake was found to be lower among older people and this requires intervention by government to redress the anomaly. Ghana's National Health Insurance Scheme (NHIS) exempts the aged from paying insurance premium. However, since majority of the respondents in this study have not attained the statutory age (70 years) to qualify for free medical services under the NHIS, they have been unable to enroll in the scheme. Pensioners of the Social Security and National Insurance Scheme (SSNIT) are also exempt from paying insurance premium. This means that rural people who have not contributed to the SNITT scheme and have not attained 70 years of age cannot receive free healthcare under the NHIS. Targeting such people remains contentious where many people have no official birthdates that can easily be verified. Therefore, the policy requires immediate review since poverty is more of a rural phenomenon. A downward review of the statutory age for exemption from paying health insurance premium based on location in a rural setting is recommended if the policy is to have the needed impact on health and human capital development, especially in rural areas.

Uptake of health insurance is positively associated with level of education. Increasing access to formal education in rural areas is therefore necessary to enhance enrolment. The popular literature lends credence to the positive effect of education on health insurance uptake. Education enhances information-seeking behavior and understanding of government policies and programs. Education also enhances quality decision making. Improving access to education in rural areas is therefore a major policy intervention that will transform most rural communities in the short and long term. It is expected that the free basic and senior high school education policies of government will give a boost to broad-based insurance uptake in the near future as the average educational level rises among the Ghanaian population. The low patronage of health insurance by women supports the popular literature that women in many developing countries are not only economically disadvantaged limiting their bargaining power in households, but also have lower education levels. Women in many developing countries are known to play major roles in economic activities including farm work, other household livelihood activities and community management, but are poorer than their male counterparts. Asset ownership, educational and income levels remain low among women, resulting in low female participation in programs and schemes such as health insurance. To address this problem, governments in developing countries and their development partners need to not only address practical women and gender needs, but implement social interventions targeting women's strategic gender needs that empower them to effectively participate in household decision-making and national life. For example, many microfinance/microcredit schemes operated by non-governmental organizations now place much emphasis on female participation in order to empower rural women. With regards to the national health insurance policy, government of Ghana should consider a lower premium for rural dwellers particularly rural women. A system whereby the rich (mostly urban dwellers) cross-subsidizes the poor (mostly rural) by paying more should be an equitable and more socially acceptable consideration among policymakers. Finally, the sensitization of the population on the importance of the scheme and the need to enroll has not achieved the expected results and further effort is therefore required in this regard. Using the local media, radio, television programs and durbars to sensitize rural dwellers will achieve higher enrolment rates.

### What is known about this topic

- The emergence of health insurance schemes in developing countries as a means to universal health coverage is well known;
- It is also known that certain socio-economic, demographic and institutional factors affect health insurance uptake;
- Patronage of health insurance remains low in most many countries, especially in developing countries.

### What this study adds

- The life cycle effect on health insurance uptake has not been examined in most studies but this was addressed in this study;
- This study modeled the interaction of age and gender, key variables affecting health insurance uptake, thereby providing a result that was hitherto unknown.

## Competing interests

The authors declare no competing interests.

## References

[1] Alhassan RK, Nketiah-Amponsah E, Arhinful DK (2016). A review of the National Health Insurance scheme in Ghana: what are the sustainability threats and prospects. PLoS ONE.

[2] Addae-Korankye A (2013). Challenges of financing health care in Ghana: the case of national health insurance scheme (NHIS). International Journal of Asian Social Science.

[3] Badu E, Agyei-Baffour P, Acheampong IO, Opoku MP, Addai-Donkor K (2018). Households sociodemographic profile as predictors of health insurance uptake and service utilization: a cross-sectional study in a municipality of Ghana. Advances in Public Health.

[4] Fox AM, Reich MR (2015). The politics of universal health coverage in low- and middle-income Countries: a framework for evaluation and action. Journal of Health Politics, Policy and Law.

[5] Kusi A, Enemark U, Hansen KS, Asante FA (2015). Refusal to enrol in Ghana's National Health Insurance Scheme: is affordability the problem. International Journal for Equity in Health.

[6] Masengeli NL, Mwaura-Tenambergen W, Mutai J, Simiyu BW (2017). Determinants of uptake of health insurance cover among Adult patients attending Bungoma County Referral Hospital. International Journal of Health Economics and Policy.

[7] Maina JM, Kithuka P, Tororei S (2016). Perceptions and uptake of health insurance for maternal care in rural Kenya: a cross-sectional study. The Pan African Medical Journal.

[8] Kimani JK, Ettarh R, Warren C, Bellows B (2014). Determinants of health insurance ownership among women in Kenya: evidence from the 2008-09 Kenya demographic and health survey. International Journal for Equity in Health.

[9] Boateng D, Awunyor-Vitor D (2013). Health insurance in Ghana: evaluation of policy holders' perceptions and factors influencing policy renewal in the Volta region. International Journal for Equity in Health.

[10] Kumi-Kyereme A, Amo-Adjei J (2013). Effects of spatial location and household wealth on health insurance subscription among women in Ghana. BMC Health Services Research.

[11] Sarpong N, Loag W, Fobil J, Meyer CG, Adu-Sarkodie Y, May J, Schwarz NG (2010). National health insurance coverage and socio-economic status in a rural district of Ghana. Tropical medicine and International Health.

[12] Kimani JK, Ettarh R, Kyobutungi C, Mberu B, Muindi K (2012). Determinants for participation in a public health insurance program among residents of urban slums in Nairobi, Kenya: results from a cross-sectional survey. BMC Health Services Research.

[13] Kiplagat I, Muriithi M, Kioko U (2013). Determinants of health insurance choice in Kenya. European Scientific Journal.

[14] Owando S (2006). Factors influencing the demand for health insurance in Kenya: a case study of Nairobi City.

[15] Ibok NI (2012). Socio-economic and demographic determinants of health insurance consumption. Canadian Social Science.

[16] Kirigia JM, Sambo LG, Nganda B, Mwabu GM, Chatora R, Mwase T (2005). Determinants of health insurance ownership among South African women. BMC Health Services Research.

[17] Amu H, Dickson KS (2016). Health insurance subscription among women in reproductive age in Ghana: do socio-demographics matter. Health Economics Review.

[18] Dror D, Radermacher R, Koren R (2007). Willingness to pay for health insurance among rural and poor persons: field evidence from seven micro health insurance units in India. Health Policy.

[19] Kirigia JM, Preker A, Carrin G, Mwikisa C, Diarra-Nama AJ (2006). An overview of health financing patterns and the way forward in the WHO African region. East African Medical Journal.

[20] Kimani D, Muthaka DI, Manda DK (2004). Healthcare financing through health insurance in Kenya: the shift to a national social health insurance scheme.

[21] Oduro-Ofori E, Aboagye AP, Acquaye NAE (2014). Effects of education on the agricultural productivity of farmers in the offinso municipality. International Journal of Development Research.

[22] Samuelson P, Samuelson W (1980). Economics.

[23] Anang BT, Dawuda I, Imoro L (2015). Determinants of savings habit among clients of Bonzali Rural Bank in the Tolon-Kumbungu District of Ghana. UDS International Journal of Development.

[24] Nyagero J, Gakure R, Keraka M, Mwangi M, Wanzala P (2012). The background, social support and behavioral characteristics associated with health insurance coverage among the older population in Kisii County, Kenya. Africa Journal of Health Sciences.

[25] Omodi DA (2009). Knowledge, perceptions and attitudes of health managers towards the proposed social health insurance scheme in Uganda. Health Policy and Development.

